# Safety and efficacy of salvage nano-particle albumin bound paclitaxel in recurrent cervical cancer: a feasibility study

**DOI:** 10.1186/s40661-016-0025-6

**Published:** 2016-04-14

**Authors:** Lindsey E. Minion, Dana M. Chase, John H. Farley, Lyndsay J. Willmott, Bradley J. Monk

**Affiliations:** Dignity Health St. Joseph’s Hospital and Medical Center, 500 West Thomas Road, Suite 660, Phoenix, AZ 85013 USA; Division of Gynecologic Oncology, Department of Obstetrics and Gynecology, University of Arizona Cancer Center at Dignity Health St. Joseph’s Hospital and Medical Center, 500 West Thomas Road, Suite 660, Phoenix, AZ 85013 USA

**Keywords:** Nano-particle albumin bound paclitaxel, Recurrent cervical cancer, Metronomic chemotherapy

## Abstract

**Background:**

After platinum and taxane chemotherapy, with or without bevacizumab, active regimens for advanced or recurrent cervical cancer are lacking. Our objective was to review a single institution experience in treating recurrent, refractory cervical cancer with nano-particle albumin bound (NAB) paclitaxel with or without bevacizumab.

**Methods:**

This retrospective case series was conducted in accordance with the regulations set forth by the Institutional Review Board at St. Joseph’s Hospital and Medical center. The chemotherapy log at the outpatient infusion center at the University of Arizona Cancer Center was reviewed to identify all advanced cervical cancer patients treated with NAB-paclitaxel from November 2011 until February 2015. The following data points were extracted from patient charts: demographic information, number of cycles, progression free survival (PFS), overall survival (OS), dose reductions and dose-limiting toxicities. In addition the average number of treatment cycles and age at recurrence were calculated.

**Results:**

A total of 12 subjects were identified as receiving treatment with NAB-paclitaxel. Mean age at time of recurrence was 47.2 years (36–55). Nine subjects had squamous cell histology and three subjects had adenocarcinoma histology. All subjects had failed treatment with platinum and taxane, or platinum and topotecan chemotherapy. Two subjects were lost to follow up. The Median number of cycles of NAB-paclitaxel was 6.5 (2–19). The total number of cycles of NAB-paclitaxel in the study population was 65. Seven subjects were treated in combination with bevacizumab. Of these, three subjects are still alive and one subject is currently receiving active treatment with NAB-paclitaxel. The median PFS and OS for all subjects that met mortality endpoint was 4.8 months and 8.9 months (*n* = 7), respectively. One subject discontinued NAB-paclitaxel secondary to peripheral neuropathy, and one subject developed a vesicovaginal fistula while obtaining combination NAB-paclitaxel and bevacizumab therapy.

**Conclusions:**

NAB-paclitaxel with or without bevacizumab is tolerable and potentially active in treating recurrent cervical cancer after failing platinum-taxane or topotecan chemotherapy. This small case series deserves confirmation through prospective clinical trials.

## Background

Hallmarks of recurrent cervical cancer continue to be poor prognosis, and limited treatment options. In this heavily pre-treated population with prior radiation, where disease is not amenable to surgical excision, there has been an evolution in cytotoxic chemotherapy regimens [1]. Gynecologic Oncology Group (GOG) protocol 204 established double therapy of cisplatin and paclitaxel as the standard of care. This combination had an overall response rate of 29.1 % [2]. Then, GOG-240 demonstrated an improvement in both PFS, and OS endpoints from this doublet with the addition of bevacizumab. By harnessing this anti-angiogenic agent, there was an addition of 3.4 months to OS [3]. However, if a GOG-240 treatment regimen fails, there are no established treatment options in recurrent cervical cancer.

Nano-particle albumin bound (NAB) paclitaxel is a 130-nanimeter, chremophor-free preparation of paclitaxel. This preparation eliminates the need for pre-medication, has a shorten infusion time, and increase tumor concentration as compared to standard preparation [4, 5].

NAB-paclitaxel was evaluated in recurrent cervical cancer a phase II trial as a part of the GOG-127 queue. For this trial all 35 subjects were taxane naïve. Subjects were treated with 125 mg/m^2^ on days 1, 8 and 15 of a 28-day cycle. Results demonstrated moderate activity with a median PFS, and OS was 5.0 and 9.4 months, respectively. Ten subjects had a partial response, and additional 15 subjects had stable disease [6].

Targeting angiogenesis, the phenotypic driver of cervical cancer, is central to effective therapy of this disease [7]. In addition to agents that directly inhibit vascular growth pathways, there is significant data corroborating that the administration of chemotherapy in reduced doses with a more frequent schedule produces an anti-angiogenic effect. So-called metronomic chemotherapy reduces endothelial repair time, endothelial cell proliferation, migration and circulating levels of endothelial progenitor cells [8–10].

Thus, based on a prior positive phase II evaluation of NAB-paclitaxel, and anti-angiogenic induced properties with metronomic administration we treated recurrent cervical cancer patient that failed prior chemotherapy with NAB-paclitaxel with or without bevacizumab.

## Methods

### Patients

This retrospective chart review was conducted in accordance with the regulations set forth by the Institutional Review Board of The Dignity Health at St. Joseph’s Hospital and Medical Center. The pharmacy chemotherapy log was review at the University of Arizona Cancer Center Chemotherapy Infusion Suite at Dignity Health at St. Joseph’s Hospital and Medical Center from to identify all patients that received nab-paclitaxel. Charts were review for patients that had the diagnoses of cervical carcinoma. All subjects were assigned a subject number, and information was de-identified during data collection. The data points collected included: demographic, tumor history, prior therapies, adverse events and treatment response.

### Statistical considerations

The primary endpoint of this series was PFS defined as initiation of nab-paclitaxel to discontinuation of treatment. Secondary endpoint included: dose reductions, completed cycles, adverse events, time to progression, time to death and indication for discontinuation of therapy. Adverse events were categorized using the Common Terminology Criteria for Adverse Events (CTCAE – version 4.0) [11]. The median number of cycles, PFS and OS were calculated.

## Results

From November 2011 to February of 2015 time period, 12 patients with the diagnoses of recurrent cervical carcinoma received NAB-paclitaxel. Two subjects were lost to follow up; one patient relocated out-of-state. The remaining 10 patients had the average age of 43.5 (range 36–55). Seven subjects were self-identified as non-Hispanic white, 4 subjects Hispanic and one other. Table 1 displays the demographic characteristic of the study group.

**Table 1:** Patient demographics

Characteristic	Category	*n*
Age Group	35–40	3
	41–45	4
	46–50	1
	51–55	3
	>56	1
Ethnicity	Non-Hispanic	7
	Hispanic	4
	Other	1
Cell Type	Squamous Cell Carcinoma	7
	Adenocarcinoma	3
Lines of Prior Therapy	1	8
	2	2
Prior Radiation	Yes	9
	No	1
Prior Surgery	Yes	5
	No	5
Prior Paclitaxel	Yes	9
	No	1
Prior Bevacizumab	Yes	6
	No	4

All subjects had recurrent cervical cancer. Of note, prior to NAB-paclitaxel therapy all subjects had failed prior cytotoxic chemotherapy. Two subjects failed 2 prior lines of chemotherapy. Chemotherapy administered in conjunction with primary radiation, as a radio-sensitizer, was not counted as a systemic chemotherapy regimen. Nine subjects had prior radiation therapy. Of the prior chemotherapy, 9 subjects had prior paclitaxel, and 6 had prior bevacizumab treatment. The only subject not treated with prior paclitaxel had failed prior topotecan. The majority of the subjects had prior pelvic radiation therapy (*n* = 9).

Subjects were treated with nab-paclitaxel with a dose of 60 mg/m^2^ to 100 mg/m^2^. There were 5 dose reductions, and 7 dose delays. Most common indication for treatment alterations was myleosuppression. Eight subjects were treated concomitantly with bevacizumab. A total of 65 cycles were completed; mean cycles completed 6.5 (range 2–19).

There were no grade 4 or 5 adverse events. The only grade 3 adverse events were anemia-requiring transfusion of packed red blood cells. One subject was diagnosed with a vesicovaginal fistula during NAB-paclitaxel therapy. For a complete list of adverse events please see Table 2.

**Table 2:** Adverse Events

	Grade 1–2	Grade 3–4
Anemia	4	6
Thrombocytopenia	1	0
Leukopenia	4	0
Fatigue	7	0
Insomnia	1	0
Weight Loss	3	0
Neuropathy	2	0
Constipation	4	0
Diarrhea	3	0
Nausea & Vomiting	5	0
Hypertension	1	0
Epistasis	1	0
Vesicovaginal Fistula	1	0
Pleural Effusion	2	0
Small Bowel Obstruction	1	0
Atrial fibrillation	1	0

**Fig. 1:**
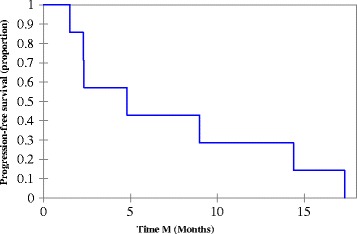
Progression free survival: Median PFS = 4.8 months (Range: 1.5-17.3 months)

**Fig. 2:**
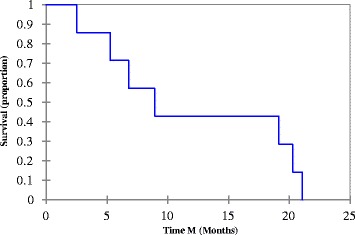
Overall survival. Median OS = 8.9 months (Range: 2.5-21.1 months)

Three subjects are still alive; one subject is currently receiving active treatment with NAB-paclitaxel. Median PFS and OS for all subjects that met mortality endpoint were 4.8 and 8.9 months (*n* = 7), respectively (Figs. 1 and 2). Median PFS and OS for the subjects treated with NAB-paclitaxel and bevacizumab that meet mortality endpoint was 6.9 and 14.02 months (*n* = 6), respectively. One subject discontinued NAB-paclitaxel secondary to peripheral neuropathy.

## Discussion

NAB-paclitaxel has three Food and Drug Administration (FDA) approved indications: locally advanced or metastatic non-small cell lung cancer, metastatic adenocarcinoma of the pancreas, and metastatic breast cancer after failure of combination chemotherapy or relapse within 6 months of adjuvant therapy [12, 13]. There have been limited evaluations of NAB-paclitaxel in cervical cancer. Here we review our experience in treating heavily pre-treated recurrent cervical cancer patients that have failed prior cytotoxic chemotherapy.

NAB-paclitaxel was previously reviewed in the 127 series. This phase II study queue was launched in the 1990’s by the GOG to evaluate recurrent squamous cell cervical cancer. Most agents studied were not effective with overall response rates ranged from 0 to 22 %. In this series, NAB-paclitaxel was the most effective agent demonstrating a partial response in 10 of 35 subjects (28.6 %). An additional 15 subjects experienced stable disease (42.9 %) [14, 15].

In comparison, within the prior phase II evaluation of NAB-paclitaxel in cervical cancer, our subjects were heavily pretreated, versus the paclitaxel naïve status of the prior study. While that data supported further investigation of NAB-paclitaxel, it is important to not disregard this significant limitation. Furthermore, data here supports the metronomic administration of this agent and this is the first report of such a dosing schedule in the setting of recurrent cervical cancer.

One subject was diagnosed with a vesicovaginal fistula during NAB-paclitaxel therapy. This subject was a 35 year old initially diagnosed with stage IIA2 adenocarcinoma. Nineteen months after her primary treatment of cisplatin and radiation, she experienced her first recurrence, and was treated with carboplatin and paclitaxel doublet therapy. Biopsy proven recurrent disease was noted during surveillance. The patient’s exam was notable for “thickened area palpable inferior to the urethra”. This subject completed 11 cycles of NAB-paclitaxel at 80 mg/m^2^ and bevacizumab 10 mg/kg, and after eleventh cycle the fistula was detected on exam.

While this subject represents 10 % of our study population, the authors argue that this is more likely a representation of a pretreated-patient with extensive pelvic disease. Furthermore, the occurrence of this complication is more likely due to the administered bevacizumab with the known complication of fistula formation. This is supported by the lack of fistulas that occurred in the phase II study of NAB-paclitaxel. As this was a limited study population, future studies will need to address the incidence of fistulas during NAB-paclitaxel therapy.

While this study provides evidence for the feasibility and tolerability of NAB-paclitaxel in the setting of recurrent cervical cancer, there are several limitations to this data including: lack of patient reported outcomes, Response Evaluation Criteria In Solid Tumors (RECIST) response, and small study population. 

## Conclusions

In conclusion, anti-angiogenic therapy has proven pivotal in the treatment of recurrent, metastatic and persistent cervical cancer, however if a GOG-240 treatment regimen fails, there are no established treatment options in recurrent cervical cancer. Metronomic-chemotherapy maybe another treatment option as this administration schedule produces anti-angiogenic properties by effecting endothelial repair time, endothelial cell proliferation, migration, and circulating levels of endothelial progenitor cells. NAB-paclitaxel was overall well tolerated with no grade 4 or 5 adverse events. NAB-paclitaxel with or without bevacizumab is feasible and potentially active in treating recurrent cervical cancer after failing platinum-taxane or topotecan chemotherapy. This small case series deserves confirmation through prospective clinical trials. 

## Abbreviations

CTCAE: common terminology criteria for adverse events
FDA: Food and Drug Administration
GOG: Gynecologic Oncology Group
mg/kg: milligram per kilogram
mg/m^2^: milligram per meters-square
NAB: nano-particle albumin bound
OS: overall survival
PFS: progression free survival
RECIST: response evaluation criteria in solid tumors
